# PIPITS: an automated pipeline for analyses of fungal internal transcribed spacer sequences from the Illumina sequencing platform

**DOI:** 10.1111/2041-210X.12399

**Published:** 2015-05-25

**Authors:** Hyun S. Gweon, Anna Oliver, Joanne Taylor, Tim Booth, Melanie Gibbs, Daniel S. Read, Robert I. Griffiths, Karsten Schonrogge

**Affiliations:** ^1^ Centre for Ecology & HydrologyMaclean BuildingBenson LaneCrowmarsh GiffordWallingford, OxonOX10 8BBUK; ^2^ Royal Botanic Garden Edinburgh20A Inverleith RowEdinburghEH3 5LRUK

**Keywords:** bioinformatics, DNA metabarcoding, fungi, internal transcribed spacer, pipeline

## Abstract

1. Studying fungal biodiversity using data generated from Illumina MiSeq sequencing platforms poses a number of bioinformatic challenges with the analysis typically involving a large number of tools for each analytical step from quality filtering to generating identified operational taxonomic unit (OTU) abundance tables.
2. Here, we introduce PIPITS, an open‐source stand‐alone suite of software for automated processing of Illumina MiSeq sequences for fungal community analysis. PIPITS exploits a number of state of the art applications to process paired‐end reads from quality filtering to producing OTU abundance tables.
3. We provide detailed descriptions of the pipeline and show its utility in the analysis of 9 396 092 sequences generated on the MiSeq platform from Illumina MiSeq.
4. PIPITS is the first automated bioinformatics pipeline dedicated for fungal ITS sequences which incorporates ITSx to extract subregions of ITS and exploits the latest RDP Classifier to classify sequences against the curated UNITE fungal data set.

## Introduction

Fungi have major ecological roles in virtually every habitat on earth, notably as decomposers, symbionts and pathogens living closely with bacteria, plants and animals. Despite their recognised functional importance, other aspects of the nature of fungi such as their diversity, distribution and ecology are much less studied than for instance that of bacteria (Desprez‐Loustau *et al*. 2007). There are an estimated 1·5–5·1 million fungal species (Hawksworth 1991; Blackwell 2011), but studying these organisms has been far from easy due to a range of factors including their complex life histories, microscopic size and cryptic features, identification problems and difficulties in circumscribing species. To add to this, traditional culturing methods are thought to only recover a small proportion of the fungi in communities sampled (O'Brien *et al*. 2005). In the last decade, however, advancement in high‐throughput sequencing brought unprecedented growth in understanding of the world of these organisms through sequencing of targeted metabarcoding marker genes directly obtained from environmental samples.

While the 16S ribosomal RNA gene has been extensively studied and exploited by researchers to describe bacterial and archaeal components of microbial communities, less effort has been devoted to the study of genetic markers to describe fungal communities. For fungi, the most widely used fungal genetic marker gene is the internal transcribed spacer (ITS) of the nuclear ribosomal subunit, which sits between the small and large subunit gene (SSU/18S and LSU/28S, respectively) (Begerow *et al*. 2010). Unlike small subunit genes, ITS shows more variability in fungi and thus is useful for inferring more specific genetic identification (Vandenkoornhuyse *et al*. 2002; Eberhardt 2010) and recently the ITS region has been formally designated as the barcode for fungal identification (Schoch *et al*. 2012). Having said that, despite being useful for inferring more specific genetic identification, its high variability means that inferring phylogenies which span distant taxonomic ranges remains a major challenge.

Processing high‐throughput data for community analysis involves a number of steps from quality filtering to taxonomic assignment, and there are computational tools specifically devoted for each step. For bacteria and archaea in particular, a wide range of open‐access, stand‐alone bioinformatics applications exist for all aspects of the analysis. Also, there are software suites which combine many of the applications to guide researchers to automate the processing of their data (Schloss *et al*. 2009; Caporaso *et al*. 2010; Angiuoli *et al*. 2011; Cole *et al*. 2014). For fungi, pipelines describing analytical steps have been published (Lindahl *et al*. 2013; Bálint *et al*. 2014), but there are only few automated pipelines and they come with notable limitations. Automated pipelines such as SCATA (http://scata.mykopat.slu.se), CLOTU (Kumar *et al*. 2011), PlutoF (Abarenkov *et al*. 2010) and ITScan (Ferro *et al*. 2014) for instance operate as a web‐based service platform and while there are clear advantages of being an online tool, there are notable disadvantages such as being dependent on the server status, number of queued jobs, bandwidth requirement, and the inability to scale operations especially given the ever increasing size of sequencing data. Furthermore, CLOTU does not make use of curated fungal barcode reference data bases such as WIU (Porras‐Alfaro *et al*. 2014) or UNITE (Kõljalg *et al*. 2013) while SCATA allows the user to upload custom reference data sets. Most notably, none of the stand‐alone applications dedicated to fungal ITS analysis to date such as FHiTINGS (Dannemiller *et al*. 2014) and CloVR‐ITS (White *et al*. 2013) incorporates the latest RDP Classifier (Wang *et al*. 2007) but rather relies on BLAST for sequence matching and taxonomic assignment. The advantage of using the RDP Classifier over BLAST is not only that it is more accurate in finding the most similar sequences (Cole *et al*. 2005; Liu *et al*. 2008), it provides a bootstrap confidence score for each of the levels of taxonomic assignment for the best matching taxa (Wang *et al*. 2007). QIIME (Caporaso *et al*. 2010), which is one of the most popular tools for processing 16S/18S ribosomal RNA genes, does offer a fungal ITS pipeline (http://qiime.org/tutorials/fungal_its_analysis.html), however, along with all of the above automated fungal‐specific pipelines, it does not extract variable ITS subregions from raw sequences. As suggested by Bengtsson‐Palme *et al*. (2013), extracting and using subregions of ITS results in a more accurate identification of species.

In this article, we present PIPITS, a user‐friendly computational tool for automated processing of large sequence data sets from quality filtering to generation of species abundance table of fungal ITS sequences. Notably, it extracts highly variable ITS subregions from raw sequences and also exploits the RDP Classifier for taxonomic assignment based on the UNITE data base and classification (See Table 1 for a comparison of the key differences between PIPITS and other existing automated pipelines dedicated for fungal ITS). PIPITS is primarily designed to analyse paired‐end reads from Illumina MiSeq sequencers, but provided that the input sequences are demultiplexed and quality checked by suitable tools such as split_libraries.py of QIIME, it can be used to process sequences from other platforms such as 454 or Ion Torrent. PIPITS, implemented in python 2.7, is open access at http://sourceforge.net/projects/pipits and is available as source code for Unix/Linux environments.

**Table 1:** A comparison of the key differences between PIPITS and other automated bioinformatics pipeline dedicated for fungal ITS sequences

Pipeline	Open‐source	Stand‐alone	Extract subregion	RDP classifier	UNITE DB
PIPITS	Yes	Yes	Yes	Yes	Yes
SCATA	Yes	Web‐based	–	–	Customisable
CLOTU	Yes	Web‐based	–	–	Customisable
PlutoF	Yes	Web‐based	–	–	Yes
ITScan	Yes	Web‐based	–	–	Customisable
CloVR‐ITS	Yes	Yes (VM)	–	–	–
FHiTINGS	Yes	Yes	–	–	Customisable
QIIME	Yes	Yes	–	Yes	Yes
UPARSE	–	Yes	–	–	Yes

## A brief description of the PIPITS pipeline

**Figure 1:**
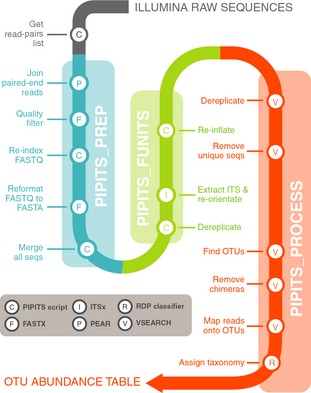
Overview/workflow of PIPITS for Illumina ITS sequences.

The PIPITS pipeline is divided into three parts namely PIPITS_PREP, PIPITS_FUNITS and PIPITS_PROCESS (Fig. 1). PIPITS_PREP prepares raw reads from Illumina MiSeq sequencers for ITS extraction; PIPITS_FUNITS extracts a fungal ITS subregion (either ITS1 or ITS2) from the reads; and PIPITS_PROCESS analyses the reads to produce operational taxonomic unit (OTU) abundance tables and the RDP taxonomic assignment table for downstream analysis. While all three steps can be run by a single command called PIPITS_ALL, it is generally recommended to run each stage separately and check the intermediate output files.

## Detailed explanation of the pipeline

### Dependencies

PIPITS depends on a number of third‐party applications, which need to be downloaded and installed:

1. vsearch (Rognes, https://github.com/torognes/vsearch/);
2. rdp classifier 2.10 or above (Wang *et al*. 2007);
3. itsx (Bengtsson‐Palme *et al*. 2013);
4. biom‐format v.1.3 (McDonald *et al*. 2012);
5. pear (Zhang *et al*. 2014); and
6. fastx‐toolkit (Hannon, http://hannonlab.cshl.edu).

### Data Set

PIPITS requires two reference data sets:

1. UNITE fungal ITS reference training data set for taxonomic assignment (http://sourceforge.net/projects/rdp-classifier/files/RDP_Classifier_TrainingData) Note that the RDP Classifier needs to be re‐trained with the data set prior to analysis and PIPITS provides a script named RETRAIN_RDP to guide the process.
2. UNITE UCHIME reference data set for chimera removal (http://unite.ut.ee/repository.php).

### PIPITS_PREP

Illumina reads are generally provided as demultiplexed FASTQ files where the Illumina machine software splits the reads into separate files, one for each barcode. PIPITS_PREP takes three mandatory inputs: first the directory with raw sequence files; second the output directory; and third a tab‐delimited file listing pairs of filenames for forward and reverse reads and the sample IDs the user wishes to use for each pair. To aid the process of making the list, PIPITS provides a script called PIPITS_GETREADPAIRSLIST which generates a tab‐delimited text file for all read‐pairs from the raw sequence directory. PIPITS_PREP begins its operation by joining read‐pairs on the overlapping regions of sequences with PEAR. The resulting assembled reads are then quality filtered with FASTQ_QUALITY_FILTER (FASTX‐Toolkit). The header of each read is then relabelled with an index number and a sample ID to minimise the size of the file. The resulting files are converted into a FASTA format with FASTQ_TO_FASTA (FASTX‐Toolkit) and merged into a single file for the next step.

### PIPITS_FUNITS

The output from PIPITS_PREP is taken as an input for this step. It is also mandatory to specify which ITS subregion (either ITS1 or ITS2) should be extracted. The script begins by dereplicating the sequences (removing redundant sequences) to shorten the processing time.

The selected subregion of fungal origin is then extracted from the sequences with ITSx and where necessary they are re‐orientated to 5′–3′ direction. It is worth noting that ITSx uses HMMER3 (Mistry *et al*. 2013) to compare input sequences against a set of models built from a number of different subregions of ITS sequences found in various organisms. This makes ITSx an ideal tool for both extraction of desired ITS subregions as well as filtering for specific groups of organisms. It also means that while PIPITS has been created with the analysis of fungal amplicons in mind, it could be adapted for the analyses of other organism groups where ITS is used as a marker by changing the ITSx settings and reference data bases. Having extracted the subregion, sequences are re‐inflated to reflect their original abundances. To date, the longest sequenceable reads from the Illumina technology are 300 bp × 2 which is not sufficient to sequence both ITS1 and ITS2 and to have an overlapping region to join them. For this reason, the program supports only a single subregion extraction mode.

### PIPITS_PROCESS

PIPITS_PROCESS makes extensive use of VSEARCH for clustering sequences into OTUs. The pipeline includes dereplicating input sequences, removing short (<100 bp) and unique sequences prior to clustering them at a user‐defined threshold (97% sequence identity by default). The resulting representative sequences for each cluster are subjected to chimera detection and removal using the UNITE UCHIME reference data set. The input ITS sequences are then mapped onto the chimera‐free representative sequences at the defined threshold, and these representatives are taxonomically assigned with the RDP Classifier against the UNITE fungal ITS reference data set. The results are then translated into two types of OTU abundance tables. In the first table, typically known as ‘OTU abundance table’, an OTU is defined as a cluster of reads with the user‐defined threshold (97% sequence identity by default), motivated by the expectation that these correspond approximately to species. In the second table, typically known as ‘phylotype abundance table’, an OTU is defined as a cluster of sequences binned into the same taxonomic assignments.

## Illustrative application example

### Sampling

Twenty samples of Scots Pine (*Pinus sylvestris*) needles were collected in June 2013 from forests and plantations in Scotland. The samples were refrigerated until processed, and this was carried out within 5 days of collection. Samples comprised 5 mm pieces from nine needles of the previous seasons growth, and these were surface sterilised as follows: 1 min 70% EtOH, 5 min 3·5% NaOCl, 30 sec 70% EtOH and 3 min 0·05% Tween 20 with ultrasonification. After reducing samples to powder with liquid nitrogen and bead beating, total DNA was extracted from replicates using both CTAB and Qiagen DNeasy Plant Mini Kit (Qiagen) methods.

### Amplicon Library Construction and Sequencing

An ITS region 2 (ITS2) gene library was constructed according to the dual indexing strategy of Kozich *et al*. (2013) utilising the fITS7 (forward) and ITS4 (reverse) primers described in Ihrmark *et al*. (2012) which anneal to the 5·8S and LSU rRNA genes flanking the ITS2 region. Briefly, each primer consisted of the appropriate Illumina adapter, an 8‐nt index sequence, a 10‐nt pad sequence, a 2‐nt linker and the gene‐specific primer. Triplicate amplicons were generated using a high‐fidelity DNA polymerase (Q5 Taq; New England Biolabs) and pooled. PCR was conducted on 10 ng of template DNA employing an initial denaturation of 30 sec at 95 °C, followed by 30 cycles of 30 sec at 95 °C, 30 sec at 52 °C and 2 min at 72 °C. A final extension of 10 min at 72 °C was also included to complete the reaction.

Amplicons were quantified using the Agilent 2200 TapeStation bioanalyser, and an equimolar pool (library) was prepared prior to purification by gel extraction (QIAEX II; Qiagen). The final concentration of the library was calculated using a SYBR green quantitative PCR (qPCR) assay with primers specific to the Illumina adapters (Kappa, Anachem).

The ITS2 library was sequenced at a concentration of 5·4 pM with a 0·6 pM addition of an Illumina generated PhiX control library. Sequencing runs, generating 2 × 300 bp, reads were performed on an Illumina MiSeq using V3 chemistry. The read 1 (R1), read 2 (R2) and index sequencing primers used were also ITS specific: R1 = sequence of the combined pad, linker and fITS7; R2 =  sequence of the combined pad, linker and ITS4; I = reverse compliment of the R2 primer (See Fig. S1).

### Data Set

Sequencing resulted in a gzipped FASTQ format file consisting of 9 396 092 paired‐end reads across the forty samples (20 using CTAB and another 20 using Qiagen). The files were placed in a directory called ‘rawdata’ in a working directory (‘$CWD’). The data used for this example is available at http://www.ebi.ac.uk/ena/data/view/PRJEB7970.

### Computing Specifications

PIPITS was tested on two Ubuntu‐based systems:

1 System 1: Bio‐Linux 7, 16‐core Intel(R) Xeon(R) CPU @ 2·27 GHz, 105 GB RAM
2 System 2: Ubuntu 14·04, a standard desktop computer, 2·93 GHz quad‐core Intel Core i3, 8GB RAM

### Dependencies and Reference Data Sets

All dependencies and reference data sets were downloaded, installed and placed in appropriate directories.

### Preparation of Re‐Trained Set of UNITE Fungal ITS Reference Data Set

Before running PIPITS, the RDP Classifier was retrained with the UNITE fungal reference training data set using RETRAIN_RDP.

Command:

retrain_rdp ‐f UNITE_sequences.fasta‐t UNITE_taxonomy.txt ‐o unite_retrained –j rdp_classifier.jar

The input directory contained the downloaded training files namely (i) taxonomy file and (ii) training sequence file with lineage. Note that this process does not need to be repeated unless a new set of training data is available to retrain the classifier.

### Editing Configuration File

The configuration file (‘pipits_config’) was then edited to direct PIPITS to the dependencies and reference data sets.

### Getting the List of Files to Process

Command:

pipits getreadpairslist ‐i rawdata/‐o readpairslist.txt

The command produced a tab‐delimited file with three columns denoting forward and reverse read filenames and sample IDs for the pairs. Note that prior to running the command, the user needs to ensure that the raw sequence filenames end with one of the following extensions: ‘.fastq’, ‘.fastq.bz2’, or, fastq.gz’. Sample IDs are taken from the first characters preceding an underscore (‘_’) from the filenames. Before proceeding to the next step, the user is encouraged to check correct filenames and desired sample IDs for the pairs are listed in the resulting file.

### PIPITS_PREP: Joining, Quality Filtering, Re‐Labelling and File‐Formatting

Command:

pipits prep ‐i rawdata ‐o out_prep ‐l readpairslist.txt

**Figure 2:**
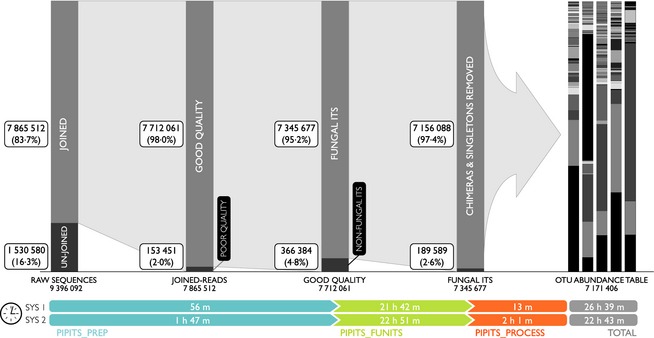
The number of sequences and time taken at each processing step. SYS1: Bio‐Linux 7, 16‐core Intel(R) Xeon(R) CPU @ 2·27GHz, 105 GB RAM; SYS2: Ubuntu 14·04, a standard desktop computer, 2·93 GHz quad‐core Intel Core i3, 8GB RAM.

9 396 092 paired‐end raw reads were processed with the command, and a FASTA file named ‘prepped.fasta’ with 7 712 061 sequences was produced (Fig. 2).

### PIPITS_FUNITS: ITS Extraction and Reads Re‐Orientation

Command:

pipits funits ‐i out_prep/prepped.fasta ‐o out_funits/‐x ITS2

The command produced a FASTA file named ‘ITS.fasta’ in ‘out_funits’ directory. The file consisted of 7 345 677 ITS2 sequences of fungal origin. The length of sequences was distributed between 101 and 461 bp with an average of 168 bp and standard deviation of 28·7 (Fig. 2). Note that this is the most time‐consuming step of the entire pipeline. It took between 22 and 23 h to process the data on both systems.

### PIPITS_PROCESS: ITS Sequences to OTU Abundance Tables

Command:

pipits process ‐i out_funits/ITS.fasta ‐o out_process/

This final step of the PIPITS pipeline took in the extracted ITS2 sequences (‘out_funits/ITS.fasta’) and produced the several output files in an output directory (‘out_process’).

The output files are:

1. assigned_taxonomy.txt
  RDP Classifier output file which lists taxonomic assignments for each OTU with bootstrap confidence score for each level of the classification hierarchy.
2. assigned_taxonomy_reformatted_filtered.txt
  Re‐formatted version of the above file (‘assigned_taxonomy.txt’) where any depth of classification with a confidence threshold of <85% is removed. This file is used to make the final OTU tables.
3. otu_table.biom, otu_table.txt
  OTU abundance tables in a BIOM and a classical tabular format
4. phylotype_table.biom, phylotype_table.txt
  Phylotype abundance tables in a BIOM and a classical tabular format
5. repseqs.fasta
  Representative sequences for OTUs.

**Figure 3:**
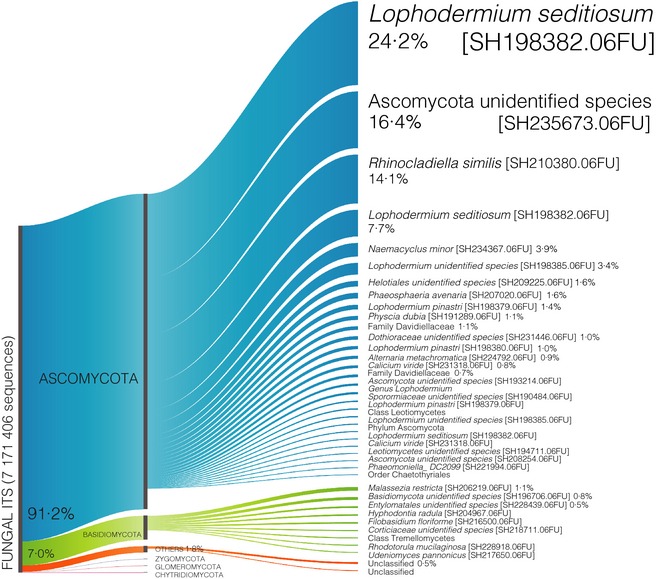
Proportion of most abundant operational taxonomic units representing 90% of the samples. In total, 91·2% of the reads (blue) were assigned to phylum Ascomycota, 7·0% (green) to phylum Basidiomycota and 0·04% to phyla belonging to Glomeromycota, Zygomycota and Chytridiomycota, while < 2% of the reads (orange) were unassignable to any of the known phylum in the data base. The three most dominant OTUs were *Lophodermium seditiosum* [SH198382.06FU] (24·2%), *Ascomycota unidentified species* [SH235673.06FU] (16·4%) and *Rhinocladiella similis* [SH210380.06FU] (14·11%).

The entire process took 22 hr 43 min and 26 hr 39 min for System 1 and System 2, respectively. After removing singletons and chimeras, 7 171 406 sequences were clustered into 1157 OTUs and 487 phylotypes (Fig. 2). In total, 91·2% of the reads were assigned to phylum Ascomycota, 7·0% to phylum Basidiomycota and 0·04% to phyla belonging to Glomeromycota, Zygomycota and Chytridiomycota while < 2% of the reads were unassignable to any of the known phylum in the data base. The three most dominant OTUs were *Lophodermium seditiosum* [SH198382.06FU] (24·2%), *Ascomycota unidentified species* [SH235673.06FU] (16·4%) and *Rhinocladiella similis* [SH210380.06FU] (14·11%) (Fig. 3).

## Discussion

**Figure 4:**
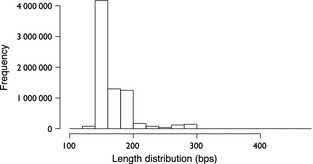
Length distribution of ITS2 sequences after extraction by PIPITS_FUNITS. Range: 101–461 bp, mean: 168, standard deviation: 28·7.

**Figure 5:**
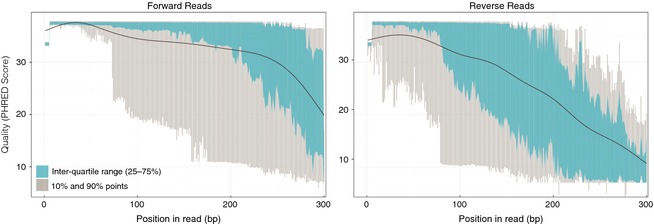
FASTQ quality scores across forward and reverse reads in one of the samples. The blue bar represents the inter‐quartile range (25–75%), the grey bar represents the 10% and 90%, and the black line represents the mean quality.

We tested PIPITS with an Illumina data set consisting of almost 10 million reads of which more than 7 million reads could be assigned to fungal species with most of the abundant phylotypes assigned to the species level. Most of the ITS2 sequences in the data set were shorter than 300 bp (Fig. 4), which was expected as the median length of fungal ITS2 was reported to be 173 bp (Nilsson *et al*. 2008). In Illumina sequencing, such fragments shorter than the insert size are subjected to ‘adapter read‐through’ where reads will contain the full length of the fragment and run into the adapter on the opposite end of the fragment in such a way that the 3′ end of the forward read will have the reverse complement of the adapter attached to the reverse read and vice versa. The paired‐end joining tool, PEAR, used by PIPITS resolves this issue by clipping the hanging regions after the joining, such that what is remaining is free of any adapter sequences. One of the advantages of using paired‐end reads even where targeted fragments are known to be short, as is the case with ITS2, is that joining Illumina paired‐end reads produces a better quality read than simply using a single read. This is because the quality of reads rapidly deteriorates nearer to the 3′ of reads as illustrated by Fig. 5.

PIPITS uses the canonical 97% sequence identity to cluster sequences into OTUs by default. While the discussion of which cut‐off threshold should be used for species‐level clustering with fungal ITS or a subregion of ITS is beyond the scope of this article, it is important to be aware that while this threshold stands for some groups of fungi, it does not hold true for others as shown by Nilsson *et al*. (2008). The study showed that intraspecific ITS variabilities in Ascomycota, Basidiomycota, Chytridiomycota, Glomeromycota and Zygomycota were shown to be 98·04%, 96·67%, 94·37%, 92·51% and 96·76%, respectively. Considering those thresholds, we expect that using 97% may potentially inflate the number of OTUs for non‐Ascomycota.

PIPITS is the first automated bioinformatics pipeline dedicated for fungal ITS sequences which incorporates ITSx to extract subregions of ITS and exploits the latest RDP Classifier to classify sequences against the curated UNITE fungal data set. One of the most useful features is that it returns taxonomically annotated OTU tables both in a classical tabular and BIOM format ready for downstream analyses. Also in contrast to other pipelines, it provides an option to output a number of intermediate files in appropriately named directories at each analytical step allowing the user to interrogate their data at any stage. PIPITS is an open‐source package available at https://sourceforge.net/projects/pipits. Detailed instructions on how to install packages and dependencies as well as setting data sets are in the user manual included in the package.

## Supporting information


**Table S1.** Duel index primer design.
